# Correspondence: The experimental requirements for a photon thermal diode

**DOI:** 10.1038/ncomms16135

**Published:** 2017-08-21

**Authors:** Bair V. Budaev

**Affiliations:** 1 Department of Mechanical Engineering, University of California, Berkeley, California 94720, USA

*Nature Communications* 8:16135 doi: 10.1038/ncomms16135 (2017); Published: 21 August 2017

In their paper, Chen *et al.*^1^ claimed the first experimental demonstration of a photon thermal diode that promises significant benefits for thermal engineering and society, such as ‘thermal regulations of building envelopes, spacecraft thermal shielding, thermal information processing’, etc. Here, however, we show that this claim is based on a fundamental error in the design and interpretation of the experiments, which cannot and do not demonstrate that the proposed device constitutes a thermal diode.

A thermal diode is a two-terminal device whose heat transfer coefficient depends on the direction of the heat flux. This property can be characterized by the inequality *Q*(*T*_1_,*T*_2_)≠−*Q*(*T*_2_,*T*_1_), if *T*_1_≠*T*_2_, where *Q*(*T*_A_,*T*_B_) is the heat flux between the terminals A and B with the temperatures *T*_A_ and *T*_B_. This phenomenon is called thermal rectification.

The essential components of the diode proposed in the paper are the collimator and the test section aligned along the axis of heat transfer, as shown in the top of Fig. 1. The collimator is made from a perforated block of absorbing material with holes parallel to the axis. The test section includes an array of pyramid mirrors with tips oriented in the axial direction. The collimator and the test section are placed between a hot blackbody cavity and the cooler side maintained at the constant temperature *T*_∞_, as shown in Fig. 1. The blackbody is heated to a certain level *T*_BBC_>*T*_∞_ and, after the system is stabilized, the heater is shut down, allowing the blackbody to cool because of thermal radiation through the collimator and the test section. The rate of change of *T*_BBC_ is recorded and is used to recover the heat flux through the system.

To demonstrate that the collimator and the test section together constitute a thermal diode it is necessary to turn this composite device around and measure the heat flux in the structure from the bottom of Fig. 1. If the pair, collimator-test section, makes the thermal diode then the cooling curves of the structures ≡◃ and ▹≡ must be different.

However, instead of considering the structure obtained by turning around the entire proposed diode, the paper 1 considers another structure, which is obtained from the ‘diode’ shown in Fig. 1 by ‘flipping the test section’, as shown in Fig. 2. Since the device in Fig. 2 is obtained by a change of the internal structure of the proposed ‘diode’ the difference in heat fluxes in the structures from Figs 1 and 2 does not represent thermal rectification, but merely confirms that different structures normally have different rates of heat transfer.

The above shows that despite an unambiguous claim that the paper^1^ presents a photon thermal diode, the reported experiments do not present a thermal diode of any kind.

## 

### Data availability

This correspondence does not use any data.

## Additional information

**How to cite this article:** Budaev, B. V. Correspondence: The experimental requirements for a photon thermal diode. *Nat. Commun.* **8**, 16135 doi: 10.1038/ncomms16135 (2017).

**Publisher’s note:** Springer Nature remains neutral with regard to jurisdictional claims in published maps and institutional affiliations.

## Figures and Tables

**Figure 1:**
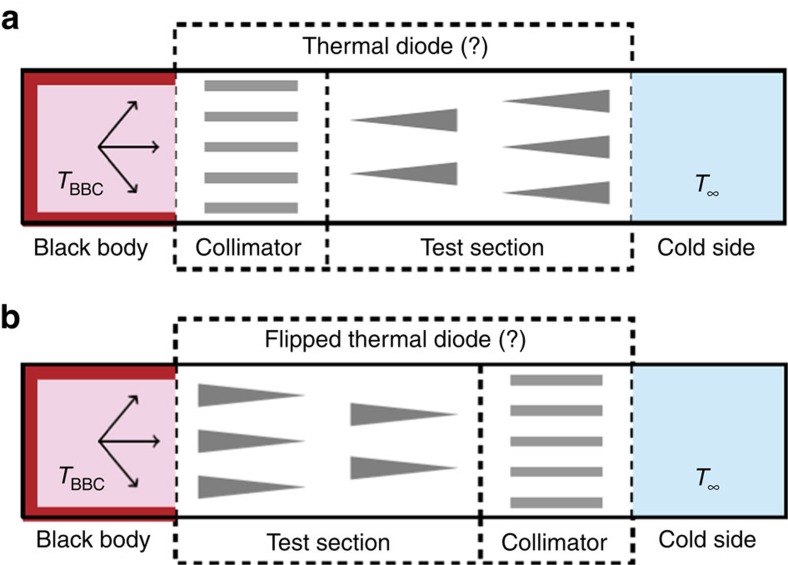
The direct and reversed bias positions of a proposed thermal diode. The proposed diode consists of the collimator and of the test section made from an array of pyramid mirrors. This diode is placed between two domains at controlled temperatures. If the direct bias position of the diode is shown in **a**, then its reverse bias position must look as in **b**.

**Figure 2:**
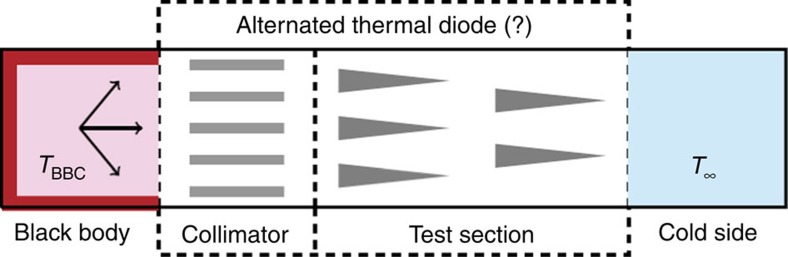
The alternated reverse bias position of the diode. The paper does not compare the thermal conductances of the proposed diode in the direct and the reverse bias positions. Instead, it considers an alternated reverse bias position obtained by turning around only a test section of the proposed diode. Since such alternation changes the the internal structure of the proposed diode, reported results do not represent thermal rectification, but merely confirm that different structures may have different rates of heat transfer.
